# Histone Deacetylase Inhibitors as a Promising Treatment Against Myocardial Infarction: A Systematic Review

**DOI:** 10.3390/jcm13247797

**Published:** 2024-12-20

**Authors:** Eduardo Sanchez-Fernandez, Sol Guerra-Ojeda, Andrea Suarez, Eva Serna, Maria D. Mauricio

**Affiliations:** 1 Department of Physiology, Universitat de Valencia, 46010 Valencia, Spain; esanfer5@alumni.uv.es (E.S.-F.); solanye.guerra@uv.es (S.G.-O.); ansuafor@alumni.uv.es (A.S.); eva.serna@uv.es (E.S.); 2 Instituto Investigación Hospital Clínico-INCLIVA, 46010 Valencia, Spain; 3 Center for Biomedical Research Network on Cardiovascular Diseases (CIBER-CV), 28029 Madrid, Spain

**Keywords:** myocardial infarction, HDAC inhibitors, I/R injury, cardiovascular protection

## Abstract

**Background/Objectives:** Acute myocardial infarction (AMI) is a critical medical condition that requires immediate attention to minimise heart damage and improve survival rates. Early identification and prompt treatment are essential to save the patient’s life. Currently, the treatment strategy focuses on restoring blood flow to the myocardium as quickly as possible. However, reperfusion activates several cellular cascades that contribute to organ dysfunction, resulting in the ischaemia/reperfusion (I/R) injury. The search for treatments against AMI and I/R injury is urgent due to the shortage of effective treatments at present. In this regard, histone deacetylase (HDAC) inhibitors emerge as a promising treatment against myocardial infarction. The objective of this systematic review is to analyse the effects of HDAC inhibitors on ventricular function, cardiac remodelling and infarct size, among other parameters, focusing on the signalling pathways that may mediate these cardiovascular effects and protect against AMI. **Methods:** Original experimental studies examining the effects of HDAC inhibitors on AMI were included in the review using the PubMed and Scopus databases. Non-experimental papers were excluded. The SYRCLE RoB tool was used to assess risk of bias and the results were summarised in a table and presented in sections according to the type of HDAC inhibitor used. **Results:** A total of 18 studies were included, 10 of them using trichostatin A (TSA) as an HDAC inhibitor and concluding that the treatment improved ventricular function, reduced infarct size, and inhibited myocardial hypertrophy and remodelling after AMI. Other HDAC inhibitors, such as suberoylanilide hydroxamic acid (SAHA), valproic acid (VPA), mocetinostat, givinostat, entinostat, apicidin, and RGFP966, were also analysed, showing antioxidant and anti-inflammatory effects, an improvement in cardiac function and remodelling, and a decrease in apoptosis, among other effects. **Conclusions:** HDAC inhibitors constitute a significant promise for the treatment of AMI due to their diverse cardioprotective effects. However, high risk of selection, performance, and detection bias in the in vivo studies means that their application in the clinical setting is still a long way off and more research is needed to better understand their benefits and possible side effects.

## 1. Introduction

Since the first definition of myocardial infarction by the World Health Organisation (WHO) in the 1950s, based mainly on the electrocardiogram, experts have agreed on up to three other definitions. The last and fourth universal definition of myocardial infarction includes evidence of acute myocardial ischaemia and the presence of myocardial injury detected by cardiac biomarkers [1].

Acute myocardial infarction (AMI) is caused by a sudden and prolonged blockage of blood flow to the myocardium because of the narrowing of the coronary arteries by atheroma plaque, thrombosis, or spasm. AMI treatment is based on restoring blood flow to the myocardium as quickly as possible, preferably by angioplasty. However, after coronary reperfusion, some signalling pathways that contribute to organ dysfunction are activated [2,3,4]. Thus, the damage is due to both ischemia and reperfusion, known as ischemia/reperfusion (I/R) injury. The search for treatments against I/R injury is necessary due to the current scarcity of effective treatments. In this regard, the inhibition of histone deacetylases (HDACs) could be a promising treatment against I/R injury [5].

HDACs catalyse the removal of acetyl groups from lysine residues on histones, thereby regulating gene expression by controlling chromatin structure. Acetylated histones promote a relaxed chromatin state that facilitates gene transcription. Conversely, deacetylated histones lead to chromatin condensation and transcriptional repression. HDACs are divided into:

- Class I: Located in the nucleus and involved in transcriptional repression. This class includes HDAC1, HDAC2, HDAC3, and HDAC8.
- Class II: Translocated between the nucleus and the cytoplasm, they regulate nuclear and cytoplasmic processes. There are two subclasses: class IIa, which includes HDAC4, HDAC5, HDAC7, and HDAC9, and class IIb, which includes HDAC6 and HDAC10.
- Class III: Called sirtuins, they are structurally and mechanically distinct from the other classes of histone deacetylases. They are NAD+ dependent and participate in the regulation of metabolism and aging.
- Class IV: Shares characteristics with classes I and II, and only HDAC11 is found within this class.

Given the cardioprotection offered by HDAC inhibition in previous studies [6,7] and the clinical need to improve outcomes after myocardial infarction, the objective of this systematic review was to analyse original research papers that have investigated the effects on ventricular function, cardiac remodelling, and infarct size, among other parameters, as well as, the mechanisms of action that could explain the cardioprotection offered by HDAC inhibitors in AMI.

## 2. Methods

This systematic review was not registered. However, it was conducted in accordance with the PRISMA 2020 statement [8] and reported following the PRISMA Checklist (See Supplementary Materials).

### 2.1. Eligibility Criteria

The following inclusion criteria were predefined for screening papers by title and abstract according to the acronym PICOS (Population, Intervention, Comparison, Outcome, and Study design) being (P): patients and AMI animal models including mice, rats and pigs; (I): administration of HDAC inhibitors; (C): control group without HDAC inhibitors; (O): studies where the outcome was clearly and convincingly presented; and (S): studies that examined the effects of the HDAC inhibitors compared with the control group. The papers included were written in English and described the cardiovascular effects of HDAC inhibitors. The exclusion criteria were: (1) publication in languages other than English or Spanish, (2) inadequacy of the data presented or poor description of the methods applied. In addition, expert opinions, editorial letters, systematic reviews, meta-analyses, case reports, and qualitative studies were removed from the review process.

### 2.2. Information Sources

The search was conducted across the PubMed and Scopus databases, and focused on papers published between January 2004 and September 2024. All studies were searched and consulted during the period from 1 July 2024 to 30 September 2024.

### 2.3. Search Strategy

All studies describing the effects of HDAC inhibitors in the treatment of AMI were identified. The search used the following combination of terms: (histone deacetylase inhibitors) OR (HDAC) AND (acute myocardial infarction).

### 2.4. Selection Process

First, the titles and abstracts of all papers were reviewed and those related to the topic of interest were selected. Next, the full manuscripts of the selected papers were obtained and those that appeared to meet the inclusion criteria were reviewed. Finally, after the exclusion of irrelevant papers, all eligible studies were formally assessed and included in this systematic review. The related data, such as the authors and time of study, experimental model, sample, HDAC inhibitor, and main results, were extracted from all the included papers. Any disagreement at the title/abstract or full manuscript screening stages was resolved by two authors independently.

### 2.5. Data Collection Process and Data Items

Each included paper was reviewed by at least two authors independently, who extracted the information using the following predetermined form: samples and models for AMI used in the study, concentrations and route of administration of the HDAC inhibitor, and the main results. Eligible outcomes included the effects of HDAC inhibitors on cardiac function, infarct size, hypertrophy, remodelling, apoptosis, inflammation, and the possible signalling pathways implicated.

### 2.6. Risk of Bias in Individual Studies

At least two authors independently assessed the risk of bias (RoB) of the included studies using SYRCLE’s RoB tool for in vivo research studies [9] and an adaptation of SYRCLE’s RoB tool [10] for in vitro research studies. Both tools contain 10 items that are related to 6 specific areas: selection bias, performance bias, detection bias, attrition bias, reporting bias, and other biases. Bias information was organised in a table with corresponding judgments: “yes” to indicate low risk of bias, ‘no’ to indicate high risk of bias, and ‘unclear’ to indicate that insufficient information was reported.

## 3. Results

### 3.1. Study Selection

**Figure 1:**
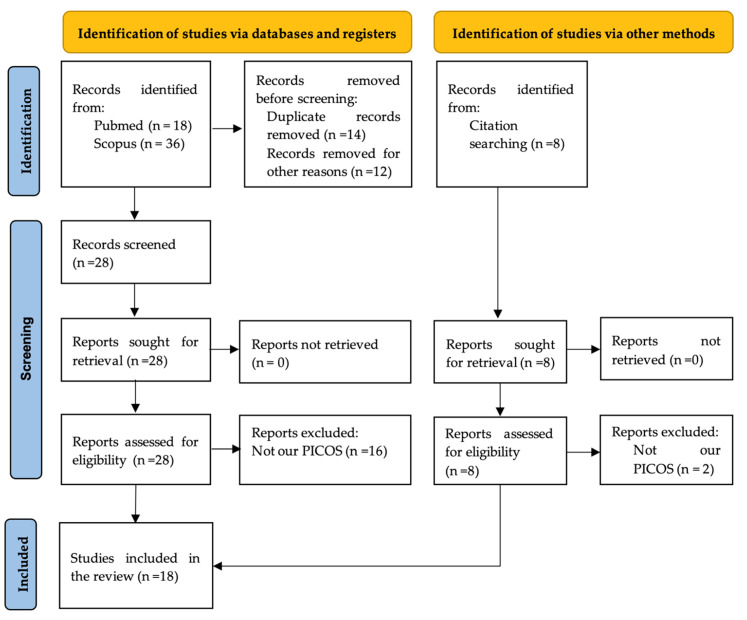
PRISMA flow diagram.

The searches retrieved 54 relevant papers, and after eliminating duplicates, a total of 28 papers were found using both databases, PubMed and Scopus. A further 16 papers were excluded because they did not meet the inclusion criteria. Next, we identified via other methods eight potentially relevant studies, of which two were excluded because they did not perform an AMI model or not used HDAC inhibitors. Finally, a total of 18 studies were included in the systematic review and evaluated on the basis of the data extracted. The complete flow chart and checklist describing the study selection process are shown in Figure 1.

### 3.2. Study Characteristics

The characteristics of the studies are shown in Section 3.4. All eligible studies were conducted between 2008 and 2024. Eleven studies (k = 11) investigated the effects of HDAC inhibitors in cells [11,12,13,14,15,16,17,18,19,20] and eighteen studies (k = 18) did so in animal models [11,12,13,14,15,16,17,18,19,20,21,22,23,24,25,26,27,28]. The most commonly used HDAC inhibitors were trichostatin A (TSA) [11,13,15,16,21,22,23,24,25,26], suberoylanilide hydroxamic acid (SAHA) [13,17], and valproic acid (VPA) [18,20,28].

### 3.3. Risk of Bias Assessment

The risk of bias assessments for each study included in this review are summarised in Table 1 and Table 2. The Modified SYRCLE’s RoB analyses indicated that all papers related to in vitro studies exhibited a high risk of bias related to selection and outcome assessment. Conversely, the SYRCLE’s RoB analyses revealed serious concerns about the high risk of selection, performance, and detection bias in in vivo studies due to the methods for grouping animals, selecting them for experimental procedures and poor blinding.

### 3.4. Results of Individual Studies

The studies included in this systematic review are summarised in Table 3 in chronological order. One of the first HDAC inhibitors to be studied was TSA, alone or in combination with other drugs, administered directly or by transplantation of TSA-treated cells; in either case, it showed a cardioprotective effect with improved ventricular function and reduced infarct size. Structurally related to TSA, suberoylanilide hydroxamic acid (SAHA) is another potential candidate for the treatment of AMI due to its ability to inhibit HDACs. Valproic acid (VPA), a branched-chain carboxylic acid, is known for its anti-epileptic effects and has recently begun to be studied for its effects as an HDAC inhibitor in the treatment of AMI. Other drugs, such as mocetinostat, givinostat, RGFP966, entinostat and apicidin, have also been studied as HDAC inhibitors and their role in the treatment of AMI is analysed in this review. To provide insights into the role of HDAC inhibitors against AMI, the studies were grouped into three sections according to the HDAC inhibitor used.

#### 3.4.1. Trichostatin A’s Effects on AMI

Trichostatin A (TSA), as a class I and class II HDAC inhibitor, has been studied in other pathological contexts, with promising results in the treatment of Alzheimer’s disease [29], cancer [30], or as a potential radiomitigator [31]. Its primary mechanism of action involves gene expression modulation by increasing histone acetylation, which can activate various signalling pathways. In this section, we summarise the signalling pathways that may be related to TSA in the context of the treatment of AMI. Zhao et al. [22], based on the hypothesis that HDAC inhibition may have similar effects to pharmacological preconditioning against I/R injury, showed that TSA improved the recovery of post-ischemic ventricular function and reduced infarct size, concluding that the increase in the activity of p38 was related to the cardioprotective mechanism of action of TSA. The p38 family of mitogen-activated protein kinases plays an important role in mediating stress-induced signalling in mammalian cells. Previous studies have shown that activation of p38 protects the heart against I/R injury [32]. However, this is not the only signalling pathway by which TSA acts, and other authors attribute different roles to TSA to explain its cardioprotective effects. Granger et al. [21] found that ischemia induces HDAC activity in the heart, leading to histone deacetylation. The authors demonstrated that TSA and scriptaid reduced infarct size by preventing ischemia-induced activation of gene programs related to hypoxia-inducible factor-1α (HIF1α), cell death, and vascular permeability. Although HIF1α activation is usually part of the cellular response to hypoxia and plays a key role (and would be positive) by inducing genes involved in angiogenesis and metabolism, its suppression by HDAC inhibitors may be beneficial in reducing the damage caused by I/R injury. That is, according to these authors, activation of HIF1α under hypoxia may be detrimental in the context of I/R, and its inhibition appears to be protective. Moreover, vascular endothelial growth factor A (VEGF-A), a key molecule that increases vascular permeability and promotes angiogenesis, is regulated by HIF1α. TSA and scriptaid blocked the increase in VEGF-A, which suggests that HDAC inhibitors may decrease ischaemic injury by down-regulating VEGF-A expression and reducing vascular permeability, potentially reducing infarct size. These results indicate that, while VEGF-A has long-term beneficial effects on angiogenesis, its acute upregulation during I/R may exacerbate injury.

On the other hand, Rajasingh et al. [11] explored the coadministration of 5-Aza-2′-deoxycytidine (Aza), an epigenetic drug that inhibits DNA methylation, and TSA to induce the conversion of bone marrow progenitor cells (BPCs) into multipotent cells (known as eiBPCs). Cardiac progenitor cells generated from eiBPCs were used for transplantation into the hearts of infarcted mice, showing improved left ventricular function after AMI, decreasing fibrosis and hypertrophy, and enhancing regeneration. It also suppressed inflammation and increased angiogenesis. In addition, they demonstrated the differentiation and proliferation of transplanted cells into cardiomyocytes and endothelial cells in situ. Although in this study the treatment did not consist of the administration of TSA directly, but rather the transplantation of cells treated with TSA, it shows the positive effects of HDAC inhibition after AMI.

The cardioprotective effects of TSA might be related to the attenuation of apoptosis, as demonstrated by Yu et al. [23] by using an I/R model in rats. Their study showed that TSA reduced myocardial infarct size, plasma lactate dehydrogenase (LDH), and creatine kinase (CK) activities, as well as the expression in the pro-apoptotic factor involved in endoplasmic reticulum stress (CHOP).

TSA also reduced active caspase-3 and stimulated Akt-1 in hearts with myocardial infarction [24], suggesting that these pathways could be involved in the increment in survival rate related to TSA. This is reinforced by the experiments carried out by Zhao et al. [25] in I/R model using MKK3^−/−^ and Akt^−/−^ mice. The authors concluded that the cardioprotective effects attributed to TSA involved MKK3 and Akt-1 signalling pathway.

One of the signalling pathways through which TSA may mediate its effects includes the FOXO3a pathway [15]. According to Guo et al. [15], TSA reduced oxidative stress markers and increased mitochondrial membrane potential, upregulated the expression of FOXO3a, superoxide dismutase (SOD) 2, and catalase, likely by increasing H4 acetylation of the FOXO3a promoter region. These findings suggest that TSA protects the myocardium from oxidative stress-mediated damage through the FOXO3a signalling pathway. These authors also found similar results to those obtained by Yu et al. [23] regarding the reduction of myocardial infarct size and activities of serum enzymes associated with myocardial injury, such as CK or LDH, in the presence of TSA.

Finally, Wang et al. [16] observed that TSA administered to mice with myocardial infarction improved cardiac remodelling and ventricular function. The cardioprotective effects attributable to TSA were related to the ability to reverse the impaired autophagic flux associated with AMI and restore autophagosomal processing of cardiac fibroblasts. TSA caused a 40% decrease in cell death.

**Figure 2:**
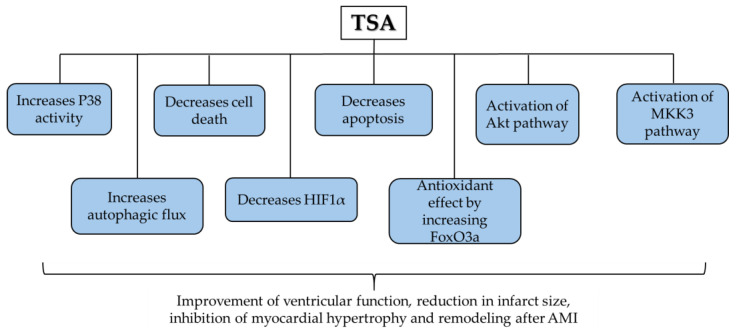
Signalling pathways involved in trichostatin A (TSA)’s effects.

In summary, TSA can reduce infarct size, restore ventricular function and prevent myocardial hypertrophy and remodelling after infarction. However, regarding angiogenesis, the role of TSA is unclear and may decrease [21] or increase it [11,24]. There could be signalling pathways involved in the beneficial effects of TSA, as we show in Figure 2.

#### 3.4.2. Suberoylanilide Hydroxamic Acid Effects on AMI

There have been few studies analysing the effects of suberoylanilide hydroxamic acid (SAHA) in the treatment of AMI. One of them was conducted by Xie et al. [13]. In that work, the authors demonstrate that both TSA and SAHA reduced infarct size and preserved systolic function in mouse and rabbit models of I/R injury. Focusing on the effects of SAHA, their in vitro experiments demonstrated that SAHA reduced cell death in neonatal rat ventricular myocytes (NRVM) subjected to I/R by inducing autophagic flux and reinforced this theory by showing that knocking out essential autophagy proteins (ATG7 or ATG5) abolished the benefit. The authors concluded that the cardioprotective effect of SAHA was linked to increased autophagic flux in the infarct border zone, which was essential for its efficacy.

After AMI, an immunological cascade is triggered that includes the recruitment of macrophages, which initially have a pro-inflammatory phenotype (type M1) characterised by the release of cytokines, such as TNF-α, IL-1β, and IL-6. Later, this phenotype changed to a reparative phenotype (type M2), with predominant release of growth factors, such as TGF-β and IL-10, promoting tissue healing and repair. Kimbrough et al. [17] demonstrated that SAHA promoted the recruitment of reparative M2 macrophages after myocardial infarction, without affecting the recruitment of inflammatory M1 macrophages until day three post-infarction. There have been few studies focusing on the effects that HDAC inhibitors could have on macrophage recruitment or more specifically on promoting the transition to M2. Therefore, more research is needed in this field, since the treatment with HDAC inhibitors could be a strategy to promote a more controlled immune response and consequently a better recovery after AMI.

#### 3.4.3. Valproic Acid Effects on AMI

Valproic acid (VPA, 2-propylpentanoic acid) is an old and well-established drug for epilepsy and other neurological diseases for which mechanisms of action are not fully understood. The proposed mechanisms for VPA include the inhibition of voltage-dependent sodium channels, which block abnormal electrical impulses and prevent seizures. In addition, VPA also increases GABA levels and activity, enhancing its inhibitory effect on the central nervous system [33]. In addition, its inhibitory effects on class I and IIa HDACs have been described in recent years, leading to its use in cancer therapy [34] and, more recently, its cardioprotective effect has been demonstrated [35], suggesting it as a promising candidate for the treatment of AMI.

VPA regulates the Foxm1 protein, which allows chromatin opening and the expression of specific genes that protect cardiomyocytes from ischaemia [18]. VPA decreased infarct size, oxidative stress, and apoptosis, likely via the Foxm1 pathway according to Tian et al. [18]. Furthermore, the authors compared the effect of VPA and SAHA on reducing infarct size at 24 h after myocardial infarction in rats and concluded that VPA better preserved cardiomyocytes and proposed that VPA, which specifically inhibits HDAC 1–3 according to these authors, was more efficient in cardiac protection than SAHA.

On the other hand, it is known that diseases such as diabetes impairs cardioprotective functions and that bone marrow-endothelial progenitor cells promote cardiac neovascularisation and attenuate ischemic injury through paracrine mechanisms involving extracellular vesicles. Based on these two assumptions, Huang et al. [20] designed a study to demonstrate that the regenerative role of extracellular vesicles could get lost in diabetes. They used two different mice models (AMI and I/R) and showed that the regenerative role of extracellular vesicles collected from the culture medium of endothelial progenitor cells was attenuated when the endothelial progenitor cells were from diabetic mice. The authors proposed that this impairment was caused partly through a HDAC-mediated epigenetic alteration, leading to functional repression in recipient cells. VPA could rescue histone 3 lysine 9 acetylation, partially recovering the extracellular vesicles’ function.

Beyond the cardioprotective effects of HDAC inhibitors, the improvement of vascular function induced by VPA has also been analysed. Very few studies have focused on vascular function; one of them, carried out by our research group, showed that VPA improves endothelial function after AMI by reversing the negative regulation of SOD 1 induced by AMI, which would increase the bioavailability of nitric oxide and the response to vasodilators such as acetylcholine [28]. Therefore, treatment with VPA could improve the reperfusion therapy after AMI.

#### 3.4.4. Cardioprotective Effects of Other HDAC Inhibitors

**Figure 3:**
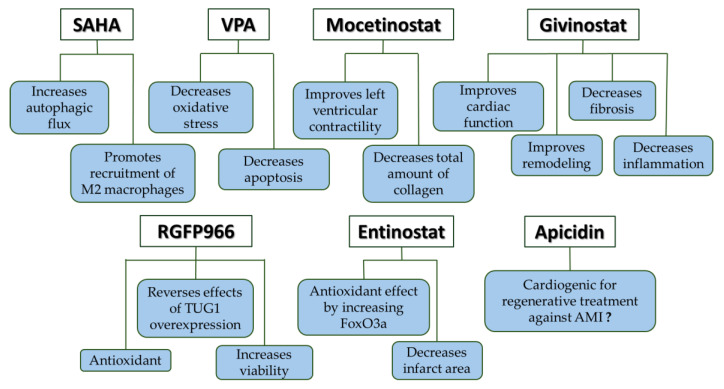
Main effects of SAHA, VPA, mocetinostat, givinostat, RGFP966, entinostat, and apicidin.

Mocetinostat, a HDAC 1, 2, and 3 inhibitor, givinostat, a class I and class II HDACs inhibitor, RGFP966, a selective HDAC3 inhibitor, entinostat, a class I and IV HDAC inhibitor, and apicidin, a class I HDAC inhibitor have also been shown to be cardioprotective. Figure 3 summarises the main effects of other inhibitors than TSA.

The HDAC1 and 2 levels are overexpressed in congestive heart failure [12], suggesting that their inhibition by mocetinostat could be beneficial in this pathology. Indeed, rats with congestive heart failure treated with mocetinostat showed improved left ventricular contractility and a reduction in the total amount of collagen without changing scar size and apoptosis [12]. However, in vitro experiments within the same study showed an increase in the levels of p21/p53 and cleaved caspase-3 in fibroblasts, indicating apoptosis in the presence of mocetinostat. Further studies are needed to delve into the mechanisms and effects that mocetinostat has on apoptosis. On the other hand, givinostat improved cardiac function and remodelling, and reduced muscle tissue loss, fibrotic area, and inflammation in a murine model of AMI [27].

Moreover, I/R promotes the expression of long noncoding RNA taurine-upregulated gene 1 (TUG1) [19]. The activation of TUG1 stimulated intracellular accumulation of ROS, increased apoptosis, decreased acetylated histone H3 lysine 9 (H3K9Ac), and increased HDAC3 expression in cardiomyocytes. Therefore, these findings suggest that TUG1 activation worsens tissue damage produced by AMI and triggers the HDAC3 signalling pathway, possibly through the TUG1/miR-132-3p/HDAC3 axis. Su Q et al. [19] showed that RGFP966 reversed the reduced viability and increased ROS production in hypoxic conditions induced by TUG1 overexpression.

Aune et al. [26] conducted a study with entinostat, TSA, and Tubastatin A in isolated heart prior to I/R. Entinostat maintained the developed pressure, pressure generation rate, pressure relaxation rate, and pressure rate product, decreased the infarct area, and increased protein expression of antioxidant enzymes through the nuclear transcription factor FOXO3a [26]. However, TSA and Tubastatin A, an HDAC6 inhibitor, did not show the same effects. Although TSA has been widely shown to have cardioprotective effects, Aune et al. [26] suggested that the lack of any beneficial effect in isolated hearts subjected to I/R was due to the ability of TSA to inhibit HDAC6 activity more effectively under their experimental conditions, an effect that could reduce the protection provided by its concurrent inhibition of class I HDACs.

Finally, some studies have relied on regenerative therapy, injecting progenitor cells treated with HDAC inhibitors instead of administering the drug directly. This is the case of the study conducted by Cho et al. [14], where apicidin-treated mesenchymal stem cells (MSCs) were intramyocardially injected into a mouse model of myocardial infarction, showing an improvement in ejection fraction and cardiac differentiation. However, it decreases the angiogenic activity of MSCs. The induction of differentiation of MSCs into cardiac lineage cells by apicidin treatment was then addressed by subsequent studies, which revealed a mechanism in which apicidin-driven cytosolic HDAC6 inhibition promoted the acetylation of yes-associated protein 1 (YAP1), allowing it to cleave from the 14-3-3 regulatory protein that retains it in the cytoplasm and be translocated to the nucleus, which transiently activates cardiac gene expression until it is recognised and degraded by the proteasome activator subunit 4 (PSME4), a subunit of the 26S proteasome. Apicidin also induces the expression of p21, which in turn promotes the transcriptional arrest of YAP1, acting in combination with PSME4 for the clearance of YAP1 [36,37]. These results imply that apicidin would also inhibit HDAC6 (class IIb HDAC) to accelerate the cardiac differentiation of MSCs. Regarding HDAC6, Aune et al. [26] suggest that its selective pharmacological inhibition would not have cardioprotective effects, or would even be harmful. Given this controversy, it is likely that HDAC6 inhibition might be ineffective as a direct treatment against AMI or I/R, while it may be useful to induce the differentiation of MSCs into cells of the cardiac lineage. Therefore, HDAC6 inhibition could be used in the context of cellular/regenerative therapy with MSCs.

## 4. Discussion

HDAC inhibitors increased ventricular function and cardiac remodelling, decreased infarct size, increased inflammation and oxidative stress, increased autophagy, decreased apoptosis, and increased endothelial function.

Owing to the limitations of the evidence included in this review, we can highlight the lack of clinical studies, probably due to the novelty of the subject. Regarding the original studies included in this review, although they all fulfilled the requirement of studying the effects of HDAC inhibitors in the treatment of AMI, in some cases, they did not explore in depth the signalling pathways affected by these inhibitors. As expected, the studies in this systematic review were not free of bias, but in all of them, the cardioprotective effect of HDAC inhibitors could be identified. However, controversy arises with the inhibition of certain HDAC isoforms, such as HDAC6. Indeed, HDAC inhibitors are not exempt from side effects, such as teratogenic effects related to an elevated state of hyperacetylation of histones [38] or neurotoxicity in zebrafish embryos [39].

HDAC inhibitors have previously been shown to be useful in various medical contexts, such as the treatment of some types of cancer [30,40] or neurodegenerative diseases [29]. However, the treatment of AMI with HDAC inhibitors is still at a preclinical stage. Therefore, the future direction should be aimed at validating these preclinical results with properly designed clinical trials that can guarantee reliable and useful results. The side effects of HDAC inhibitors in humans will depend on the dosing regimen and health status of the patient, and will need to be analysed in future clinical trials. Further preclinical and clinical studies will help us to better understand their benefits and possible side effects.

In conclusion, this systematic review provides a potential and promising application of HDAC inhibitors in the treatment of AMI.

## Figures and Tables

**Table 1:** Risk of bias for in vitro studies.

Study	Item 1	Item 2	Item 3	Item 4	Item 5	Item 6	Item 7	Item 8	Item 9	Item 10
Granger et al., 2008 [21]	No	Yes	Yes	Yes	Yes	No	No	Yes	Yes	Yes
Rajasingh et al., 2011 [11]	No	Yes	Yes	Yes	Unclear	Yes	No	No	Yes	Yes
Nural-Guvener et al., 2014 [12]	No	Yes	Yes	Yes	Yes	Yes	No	Yes	Yes	Yes
Xie et al., 2014 [13]	No	Yes	Yes	Yes	Yes	Yes	No	Yes	Yes	Yes
Cho et al., 2017 [14]	No	Yes	Yes	Yes	Yes	Yes	No	No	Unclear	Unclear
Guo et al., 2017 [15]	No	Yes	Yes	Yes	Yes	Yes	No	Yes	Yes	Yes
Wang et al., 2018 [16]	No	Yes	Yes	Yes	No	Yes	No	No	Unclear	Unclear
Kimbrough et al., 2018 [17]	No	Yes	Yes	Yes	Unclear	Yes	No	Yes	Unclear	Unclear
Tian et al., 2018 [18]	No	Yes	Yes	Yes	Yes	Yes	No	Yes	Yes	Yes
Su et al., 2020 [19]	No	Yes	Yes	Yes	Yes	Yes	No	Yes	Yes	Yes
Huang et al., 2022 [20]	No	Yes	Yes	Yes	Yes	Yes	No	Yes	Yes	Yes

**Table 2:** Risk of bias for in vivo studies.

Study	Item 1	Item 2	Item 3	Item 4	Item 5	Item 6	Item 7	Item 8	Item 9	Item 10
Zhao et al., 2007 [22]	No	Yes	No	Unclear	No	Yes	No	Yes	Yes	Yes
Granger et al., 2008 [21]	No	No	No	No	No	No	No	Unclear	Yes	Yes
Rajasingh et al., 2011 [11]	No	No	No	No	No	No	No	Unclear	Yes	Yes
Ling Yu et al., 2012 [23]	No	Unclear	No	No	No	No	No	Unclear	Yes	Yes
Zhang et al., 2012 [24]	No	Unclear	No	No	No	No	No	Unclear	Yes	Yes
Zhao et al., 2013 [25]	No	Unclear	No	Unclear	No	Yes	No	Unclear	Yes	Yes
Nural-Guvener et al., 2014 [12]	No	Yes	No	No	No	No	No	Yes	Yes	Yes
Xie et al., 2014 [13]	No	Unclear	No	No	No	No	No	Unclear	Yes	Yes
Aune et al., 2014 [26]	No	Unclear	No	No	No	No	No	Unclear	Yes	Yes
Cho et al., 2017 [14]	No	Yes	No	Unclear	No	Yes	No	No	Unclear	Unclear
Guo et al., 2017 [15]	No	Yes	No	No	No	No	No	Yes	Yes	Yes
Wang et al., 2018 [16]	No	Unclear	No	No	No	No	No	Unclear	Unclear	Unclear
Milan et al., 2018 [27]	No	Yes	No	No	No	No	No	Yes	Yes	Yes
Kimbrough et al., 2018 [17]	No	No	No	No	No	No	No	Unclear	Unclear	Unclear
Tian et al., 2018 [18]	No	No	No	Unclear	No	Yes	No	Unclear	Yes	Yes
Su et al., 2020 [19]	No	Yes	No	Unclear	No	Yes	No	No	Yes	Yes
Huang et al., 2022 [20]	No	Unclear	No	No	No	No	No	Unclear	Yes	Yes
Guerra-Ojeda el al., 2024 [28]	No	Yes	No	No	No	No	No	Yes	Yes	Yes

**Table 3:** List of selected studies in chronological order.

Authors	Samples and Models	HDAC Inhibitor, Concentrations, and Route of Administration	Main Results
Zhao et al., 2007 [22]	Isolated mouse heart subjected to I/R. Pharmacological preconditioning with TSA	TSA (50 nM) in isolated heart in early preconditioning and TSA (0.1 mg/kg, i.p.) in delayed preconditioning	TSA improved post-ischemic ventricular function and reduced infarct size in both types of preconditioning. The protective mechanism of TSA is through elevation of p38 activity
Granger et al., 2008 [21]	8-week-old CD-1 mice subjected to I/R	TSA (1 μg/g i.p.) Scriptaid (1 μg/g i.p.)	Both TSA and Scriptaid reduced the size of myocardial infarction by more than 50%. Both TSA and Scriptaid prevented ischemia-induced activation of gene programs related to hypoxia-inducible factor-1α, cell death, and vascular permeability. HDAC4 was identified as a key mediator in the protective effects of HDACIs on ischemic cardiac myocytes
	In vitro: Neonatal cardiac myocytes isolated from 1-day-old CD-1 mouse pups in hypoxic conditions	In vitro: TSA: 100 nmol/L Scriptaid: 6 nmol/L	
Rajasingh et al., 2011 [11]	8-week-old male C57BL/6J mice with AMI (ligation of left anterior descending coronary artery) for 28 days	Intramyocardial injection of cardiac progenitor cells treated with TSA and Aza	TSA + Aza improved left ventricular function, decreased fibrosis and hypertrophy, enhanced regeneration, suppressed inflammation and increased angiogenesis
	Bone marrow progenitor cells	25 nM TSA + 50 nM Aza (DNA methylation inhibitor)	
Ling Yu et al., 2012 [23]	Male Wistar rats. I/R model by occlusion/release of the left anterior descending coronary artery (30 min/24 h)	TSA (0.05, 0.1 and 0.2 mg/kg/day i.p.) for 5 days	TSA decreased CHOP-induced apoptosis and contributed to cardioprotection
Zhang et al., 2012 [24]	Heart of 10–12-week-old male ICR mice. AMI model (permanent ligation of the left anterior descending artery)	TSA (0.1 mg/kg daily i.p.) for 8 weeks after AMI	TSA treatment improved ventricular function, cardiac endogenous regeneration, and angiogenesis. Inhibits myocardial hypertrophy and remodelling
Zhao et al., 2013 [25]	Hearts of adult male B6.129 wild-type mice, MKK3−^/^− mice and Akt-1−^/^− I/R model	TSA (0.1 mg/kg i.p. 24 h) before sacrifice	HDAC inhibition by TSA in I/R injury induced cardioprotective effects through the stimulation of the MKK3/Akt-1 pathway
Nural-Guvener et al., 2014 [12]	Rats with congestive heart failure after AMI (left coronary artery ligation)	Mocetinostat (10 mg/kg daily) for 3 weeks (starting 3 weeks post-surgery)	Mocetinostat improved left ventricular contractility and reduced the amount of collagen
	In vitro: Isolated atrial or ventricular CD90+/cKit- cardiac fibroblasts	In vitro: 1 μM and 2 μM of Mocetinostat for 7 days	In vitro: Mocetinostat increased the levels of p21/p53 and cleaved caspase-3 in atrial fibroblasts
Xie et al., 2014 [13]	8–12-week-old C57BL6 wild-type mice subjected to I/R (45 min ischemia and 2 h reperfusion) and RFP-GFP-LC3 transgene mice	TSA (1 mg/kg i.p.) one day before I/R. SAHA (30 mg/kg or 50 mg/kg s.q. (pretreatment group) or 100 mg/kg s.q. (reperfusion-only group)	TSA reduced infarct size by around 50%. SAHA (50 mg/kg) reduced infarct size by around 45%, and partially preserved systolic function after I/R. SAHA (50 mg/kg s.q.) increased autophagic flux in the infarct border zone in RFP-GFP-LC3 transgene mice
	New Zealand White rabbits subjected to I/R	SAHA (150 mg/kg s.q. pretreatment group or 300 mg/kg s.q. reperfusion-only group)	Both the pretreatment and reperfusion-only treatment with SAHA reduced infarct size. SAHA treatment mitigated the decrease in systolic function after I/R. LC3-II levels increased and apoptosis decreased in the infarct border zone of SAHA-treated hearts
	In vitro: NRVM subjected to I/R. ARVM subjected to I/R	In vitro: SAHA (2 μM)	In vitro: SAHA induced autophagic flux and decreased I/R-induced cell death in NRVM and ARVM. Knockdown of essential autophagy proteins (ATG7 or ATG5) abolished the protective effect of SAHA
Aune et al., 2014 [26]	Isolated rat heart subjected to ischemia/reperfusion (30 min/120 min)	TSA (1.0 mg/kg i.p.), entinostat, (10 mg/kg i.p.) and tubastatin A (10 mg/kg i.p.) administrated to rat for 24 h and again 1 h before heart excision	Entinostat preserved left ventricular function and reduces infarct size. Entinostat increased the expression of SOD2, catalase, and nuclear FOXO3a
Cho et al., 2017 [14]	7–8-week-old male inbred Balb/C nude mice. MI model (proximal left anterior descending coronary artery ligation). In vitro: MSCs	Intramyocardial injection of MSCs treated with 3 μM apicidin for 24 h for 7 days	Combined treatment with non-treated and apicidin-treated MSCs constitutes an optimised regenerative therapy approach against MI as it combines the cardiogenic properties of apicidin treatment of mesenchymal stem cells while maintaing angiogenic activity of non-treated MSCs
Guo et al., 2017 [15]	Rats subjected to I/R	TSA (0.2, 0.1 and 0.05 mg/kg i.p.) once daily for 5 days before undergoing I/R surgery	TSA reduced myocardial infarct size and decreased the activities of lactate dehydrogenase, aspartate aminotransferase, and creatine kinase in rats. TSA decreased malondialdehyde levels and increased SOD activities in myocardial tissue
	In vitro: Cell Model: H9c2 rat myocardial cell line	In vitro: TSA (50 nmol/L) 1 h of incubation	In vitro: TSA improved the viability of H9c2 cells exposed to H_2_O_2_, reduced intracellular ROS levels, increased the mitochondrial membrane, and increased the expression of FOXO3a, SOD2, and catalase, which are linked to increased H4 acetylation of the FOXO3a promoter region
Wang et al., 2018 [16]	MI induced by ligation of the left anterior descending (LAD) coronary artery in C57/BL mice. Cultured neonatal rat cardiac fibroblasts	TSA (0.1 mg/kg/day i.p.)	TSA prevents cardiac remodelling after myocardial infarction with a mechanism of action involving autophagosomal processing of cardiac fibroblasts
Milan et al., 2018 [27]	10-week-old female C57BL/6 wild-type mice with AMI (through permanent ligation of the left descending coronary artery)	Givinostat (10 mg/kg/day i.p.) for 1, 3, 7, 15 or 30 days	Givinostat improved cardiac function, remodelling, and reduced loss of muscle tissue, cardiac fibrotic area, and inflammation
Kimbrough et al., 2018 [17]	Hearts of 12–15-week-old CD1 male mice with AMI (induced by left anterior descending coronary artery permanent ligation)	SAHA. 1st dose immediately after surgery (100 mg/kg i.p.). Subsequent doses in drinking water	SAHA promotes the recruitment of reparative M2 macrophages after AMI. SAHA promotes angiogenesis, and preserves ventricular function after AMI
	In vitro: RAW 264.7 murine macrophages and murine bone marrow macrophages (isolated from 8–10-week-old CD1 mice femur and tibia)	SAHA at 5 µM and TSA at 100 nM	HDAC inhibition induces M1 to M2 polarisation in cultured macrophages
Tian et al., 2018 [18]	I/R model in rats MI in mice (permanent occlusion of the left descending coronary artery)	VPA (250 mg/kg i.p.)	VPA improved cardiac function after AMI and reduced infarct size, both in the short and long term. VPA decreased oxidative stress and apoptosis. VPA mechanism involves Foxm1 upregulation
	In vitro: Human embryonic stem cells derived cardiomyocytes in hypoxia-reperfusion model	In vitro: VPA (10 µM)	
Su et al., 2020 [19]	Heart isolated from I/R induced AMI mice of 10-week-old through occlusion of the left anterior descending coronary artery for 0, 30, and 60 min	---	Taurin gene 1 (TUG1) level increased with increasing ischemia time in infarcted tissues. siTUG1 infection before I/R reduced infarct size, reduced ROS production, inhibited apoptosis, and down-regulated HDAC3 expression
	In vitro: Primary cardiomyocytes isolated from neonatal C57BL/6 mice (1–3 day-old), under hypoxia	HDAC3 inhibitor (RGFP966,10 uM)	In vitro: siTUG1 improved viability, reduced ROS production and downregulate HDAC3 expression in hypoxia. TUG1 overexpression further reduced the viability and increased ROS production in hypoxic conditions, which was reversed by the HDAC3 inhibitor
Huang et al., 2022 [20]	10–12-week-old male mice with AMI (left coronary artery ligation) or I/R model	Intramyocardial injection of endothelial progenitor cells treated with VPA (2 × 10^5^ cells)	VPA rescues the cardioprotective effects of extracellular vesicles from endothelial progenitor cells in cardiac injury treatments through stem cell therapy, lost in diabetes, by reversing histone 3 lysine 9 acetylation–deacetylation
	In vitro: endothelial progenitor cells from 8–10-week-old diabetic and non-diabetic male mice	VPA (1 mM) 24 h	
Guerra-Ojeda el al., 2024 [28]	Abdominal aorta of 15-week-old male New Zealand White rabbits with AMI (induced by occlusion of the left circumflex coronary artery for 1 h followed by reperfusion)	VPA (500 mg/kg/day s.q.) for 5 weeks	AMI caused endothelial dysfunction in the abdominal aorta, which did not occur in the presence of VPA. The mechanism by which VPA acted is related to a vascular antioxidant effect, which increases NO bioavailability and vasodilation, helping in reperfusion therapy

I/R: ischemia/reperfusion; TSA: Trichostatin A; i.p.: intraperitoneally; AMI: acute myocardial infarction; MI: myocardial infarction; AZA: 5-Aza-2′-deoxycytidine; s.q.: subcutaneusly; MKK3: Mitogen-activated protein kinase kinase 3; SAHA: suberoylanilide hydroxamic acid; NRVM: neonatal rat ventricular myocytes; ARVM: adult rat ventricular myocytes; MSCs: immortalised human bone marrow-derived mesenchymal stem cells; VPA: valproic acid; SOD: superoxide dismutase; MI: myocardial infarction; ^−/−^: null homozygous state (knock out); NO: nitric oxide.

## Supplementary Materials

The following supporting information can be downloaded at: https://www.mdpi.com/article/10.3390/jcm13247797/s1, PRISMA 2020 Checklist.
